# An effective sequence-alignment-free superpositioning of pairwise or multiple structures with missing data

**DOI:** 10.1186/s13015-016-0079-3

**Published:** 2016-06-21

**Authors:** Jianbo Lu, Guoliang Xu, Shihua Zhang, Benzhuo Lu

**Affiliations:** Human Genetics Resource Center, National Research Institute for Family Planning, Beijing, 100081 China; Graduate School of Peking Union Medical College, Beijing, 100730 China; National Center for Mathematics and Interdisciplinary Sciences, Academy of Mathematics and Systems Science, Chinese Academy of Sciences, Beijing, 100190 China

**Keywords:** Superposition, Protein structure alignment, Iterative closest point

## Abstract

**Background:**

Superpositioning is an important problem in structural biology. Determining an optimal superposition requires a one-to-one correspondence between the atoms of two proteins structures. However, in practice, some atoms are missing from their original structures. Current superposition implementations address the missing data crudely by ignoring such atoms from their structures.

**Results:**

In this paper, we propose an effective method for superpositioning pairwise and multiple structures without sequence alignment. It is a two-stage procedure including data reduction and data registration.

**Conclusions:**

Numerical experiments demonstrated that our method is effective and efficient. The code package of protein structure superposition method for addressing the cases with missing data is implemented by MATLAB, and it is freely available from: http://sourceforge.net/projects/pssm123/files/?source=navbar

**Electronic supplementary material:**

The online version of this article (doi:10.1186/s13015-016-0079-3) contains supplementary material, which is available to authorized users.

## Background

Superposition is a frequently used method to measure spatial similarity of three-dimensional objects such as computer vision, image science and molecular biology. Molecular biology employs superposition to support a wide variety of tasks. It is a very important problem to superimpose two or more protein structures in structural bioinformatics. Superpositioning problems have been explored by many studies [1–5]. The optimal superposition of three-dimensional (3D) conformations of similar structures is necessary in many real cases. Determining an optimal superposition normally requires a one-to-one correspondence between the atoms in the different structures [6]. The superposition of multiple structures’ situation is complicated by the fact that if structure X is superimposed on structure Y and structure Z is superimposed on structure Y, then, in general, structure X is not optimally superimposed on structure Z. In this case, the superposition of X on Z is only optimal superposition if two of the three structures are identical in shape.

A superposition is a particular orientation of objects in three-dimensional space. There are many approaches to solve this problem. One of the approaches to solve the superpositioning problem is the method proposed by Kabsch [3], which allows computing the optimal transformation via singular value decomposition of a covariance matrix derived from the coordinates of the corresponding three-dimensional structure. Another approach for this problem proposed by Kearsley [7] uses the algebra of quaternions. Multiple structure superposition programs have many applications, including understanding evolutionary conservation and divergence, functional prediction, automated docking, comparative modeling, protein and ligand design, construction of benchmark data sets and protein structure prediction and so on [8–11].

Structure alignment is different from superposition of structures. A structural alignment is the assignment of amino acid residue-residue correspondences between similar structural proteins [12]. One way to represent an alignment is using the familiar row and column matrix format, in which sequence alignments use single letter abbreviations for residues. Alignments of amino acid sequences of proteins play important roles in structure molecular biology such as the study of evolution in protein families, the identification of patterns of conservation in sequences, homology modeling, and protein crystal structure solution by molecular replacement.

In molecular biology, corresponding residues have similar structures. Many homologous proteins share a common core structure, in which the chain retains the topology of its folding pattern, but varies in geometric details. This retained similarity makes it possible to align the residues of the core. Since the structure of many proteins is still unknown and proteins with similar structural motifs often exhibit similar biological properties even when they are distantly related, structure alignment can help characterize the role of many proteins.

There are two ways for protein structure alignments, sequence-based alignments and non-sequence-based alignments (i.e. Structal [13], TM-align [13], LovoAlign [13]). For closely related proteins, sequence-based alignments give consistent answers, reflecting evolutionary divergence. For distantly related proteins, however, sequence-based alignments lead to diverse residue correspondences. At this case, we need non-sequence-based alignments. Non-sequential alignments can handle many cases such as reordering of domains and circular permutations [13–15].

Most multiple structure alignment programs are based on pairwise structural alignment programs [16, 17]. Even simplified variants of structure alignment are known to be NP-hard [18, 19]. In many cases, certain residues are missing. For example, one crystal structure of a protein may omit loop regions that are present in another crystal structure of the same protein [20]. Most of the multiple structural alignment methods divide it into two subproblems. The first is to identify multiple corresponding structural elements. The second is to calculate the appropriate rigid-body transformation for each structure to create an optimal superposition.

There are three broad classes for structure alignment programs: the first class is aligned fragment pair (AFP) chaining methods [21]. The second class [22], is distance matrix methods. The third class includes everything else, such as geometric hashing and methods using secondary structural elements [22]. THESEUS is a software to consider the missing data by adopting an expectation-maximization (EM) algorithm [23]. However, EM algorithm relies on a sequential structure alignment and it is highly dependent on the choice of the initial value. In this paper, we propose a new method for non-sequential structure superposition. We use the combination of principal component analysis (PCA) and iterative closest point (ICP) registration techniques. The point of our method is we treat the proteins as the whole structures.

In this work, we propose a simple and efficient protein structure superposition method for addressing the cases with missing data (PSSM). We adopt a two-stage procedure including data reduction and registration techniques to address this problem. We have applied it to the cytochrome C data, Globins family data, Serine Proteinases family data, Fisher’s dataset and the simulated data to demonstrate its efficiency and accuracy.

## Methods

**Fig. 1:**
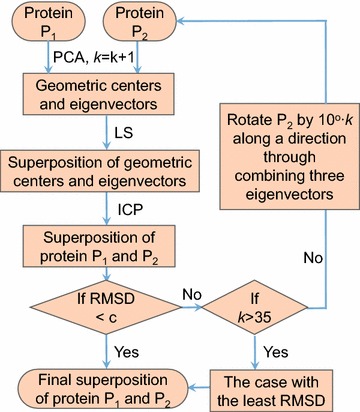
The flow chart of our pairwise protein structure superposition algorithm for missing data (PSSM)

Here we introduce a two-stage method for the optimal superposition of pairwise and multiple structures with incomplete data. In the first stage, the key is to adopt a data reduction technique to get a reduced representation which is not sensitive to the noise and the missing residues. Based on the representation, we can obtain a rough superposition of pairwise or multiple structures with a least square technique. In the second stage, we employ the powerful iterative closest point (ICP) algorithm to further refine the superposition and find the optimal solution (Fig. 1).

The iterative closest point algorithm, originally introduced in the area of computer vision for image registration, can be used in bioinformatics for the alignment of complete protein structures. Bertolazzi [24] used this method for the structural alignment of protein surfaces.

We implemented the method in Matlab software as a package named PSSM.

### Discovering rough superpositioning based on principal-axes transform

In this section we introduce the principal component analysis, the principal-axes transform techniques and the rotational search needed for some cases.

### principal component analysis

Principal component analysis (PCA) is a very popular subspace analysis technique which is successfully applied in many domains for dimension reduction. It helps you reduce the number of variables in an analysis by describing a series of uncorrelated linear combinations of the variables that contain most of the variance. This reduction is achieved by transforming the original variables to the uncorrelated principal components—new variables. This new variables are ordered so that the first few ones keep the most of the variation in all of the original variables.

The computation of principal components can give the principal component of the points. Then, we rotate the points along this principal component. This allows us to get the best initial value of the points. After this step, we employ the iterative closest point algorithm to further refine the superposition and find the optimal solution.

### Principal-axes transform

**Fig. 2:**
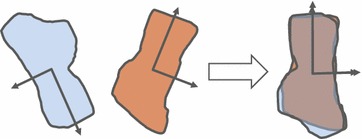
Illustration of the principal axes transform for aligning two two-dimensional shapes

The principal axes of a protein structure are computed directly from its atomic coordinates. The first moment of these points is their center of mass, and the three eigenvectors and eigenvalues of the second moment tensor give the principal axes and their relative lengths. The transform aligns the centers of mass and principal axes in order of decreasing relative lengths. The principal axes are coarse shape descriptors and are affected very little by noise or small differences in the structure and region being aligned [13]. The least square method was used to align corresponding principal-axes. As an example, we demonstrated the alignment of two two-dimensional shapes using the principal-axes transform in Fig. 2.

### Rotational search strategy

The principal-axes transform is expected to yield correct rough superpositioning for many initial values. However, it may fail to produce proper ones in some cases. We consider a rotational search strategy to improve this situation to test multiple orientations. The axis of rotation is a line which goes through points (0, 0, 0) (geometric center) and *u* (the linear combination of eigenvectors of one protein). The interval degree is set as $10^{\circ }$. In practice, the principal-axes alignment method is applied first, followed by a rotational search if the resulting structure superpositioning does not give satisfactory results below a given RMSD (root mean squared deviation) value, then the principal-axes alignment method is applied again.

### Structures with random rotations

To show the effectiveness of PSSM method, we use random rotational matrices to generate a random corresponding structure. A random rotational orthogonal matrix is generated by a MATLAB function [i.e., orth(rand(3,3))]. As we know, the rotational matrices change the points’ position and orientation.

### Refining the superpositioning based on iterative closest point algorithm

The iterative closest point (ICP) algorithm is based on quaternion [25]. The unit quaternion is a four vector $\vec {q_R}=[q_0,q_1,q_2,q_3]^T$, where $q_0 \ge 0$, and $q_0^2+q_1^2+q_2^2+q_3^2=1$. The $3\times 3$ rotation matrix generated by a unit rotation quaternion is

$$\begin{aligned} R(\vec {q_R})= \left[ \begin{array}{lll} q_0^2+q_1^2-q_2^2-q_3^2 &{} 2(q_1q_2-q_0q_3) &{} 2(q_1q_3+q_0q_2) \\ 2(q_1q_2+q_0q_3) &{} q_0^2+q_2^2-q_1^2-q_3^2 &{} 2(q_2q_3-q_0q_1) \\ 2(q_1q_3-q_0q_2) &{} 2(q_2q_3+q_0q_1) &{} q_0^2+q_3^2-q_1^2-q_2^2 \end{array}\right] . \end{aligned}$$

Let $\vec {q_T}=[q_4,q_5,q_6]^T$ be a translation vector. The complete registration state vector $\vec {q}$ is denoted as $\vec {q}=[\vec {q_R},\vec {q_T}]^T.$ Let $P=\{{\vec p_i}\}_{i=1}^{N_p}$ be a measured data point set to be aligned with a model point set $X=\{{\vec x_i}\}_{i=1}^{N_x}$, where $N_x=N_p$ and each point $\vec p_i$ corresponds to the point $\vec x_i$ with the same index. The mean square objective function to be minimized is

1 $$\begin{aligned} f(\vec q)=\frac{1}{N_p}\sum _{i=1}^{N_p}||\vec {x_i}-R(\vec {q_R})\vec {p_i}-\vec {q_T}||^2. \end{aligned}$$

Defining $\vec {\mu _p}$ and $\vec {\mu _x}$ by:

2 $$\begin{aligned} \vec {\mu _p}=\frac{1}{N_p} \sum _{i=1}^{N_p}\vec {p_i}, \end{aligned}$$

3 $$\begin{aligned} \vec {\mu _x}=\frac{1}{N_x} \sum _{i=1}^{N_x}\vec {x_i}, \end{aligned}$$

The cross-covariance matrix $\Sigma _{px}$ of the sets *P* and *X* is given by

4 $$\begin{aligned} \Sigma _{px}=\frac{1}{N_p} \sum _{i=1}^{N_p}[(\vec {p_i}-\vec {\mu _p})(\vec {x_i}-\vec {\mu _x})^T]=\frac{1}{N_p} \sum _{i=1}^{N_p}[\vec {p_i}\vec {x_i}^T]-\vec {\mu _p}\vec {\mu _x}^T. \end{aligned}$$

The symmetric $4\times 4$ matrix $Q(\Sigma _{px})$ is:

$$\begin{aligned} Q(\Sigma _{px})= \left[ \begin{array}{cc} tr(\Sigma _{px}) &{} \Delta ^T \\ \Delta &{} \Sigma _{px}+\Sigma _{px}^T-tr(\Sigma _{px})I_3 \end{array}\right] , \end{aligned}$$

where $\Delta =[A_{23} A_{31} A_{12}]^T$ and $A_{i,j}=(\Sigma _{px}-\Sigma _{px}^{T})_{i,j}$. $I_3$ is the $3\times 3$ identity matrix. The unit eigenvector, denoted as $\vec {q_R}=[q_0,q_1,q_2,q_3]^T$, corresponding to the maximum eigenvalue of the matrix $Q(\Sigma _{px})$ is selected as the optimal rotation. The optimal translation vector is given by

5 $$\begin{aligned} \vec {q_T}=\vec {\mu _x}-R(\vec {q_R})\vec {\mu _p}. \end{aligned}$$

This least square quaternion operation is $O(N_p)$ and is denoted as

6 $$\begin{aligned} (\vec {q},d_{ms})=\mathcal {Q}(P,X), \end{aligned}$$

where $d_{ms}$ is the mean square point matching error. The notation $\vec {q}(P)$ is used to denote the point set *P* after transformation by the registration vector $\vec {q}$.

Let *d* be the distance metric between an individual data point $\vec {p}$ and a model shape *X*, then $d(\vec {p},X)$ will be denoted:

7 $$\begin{aligned} d(\vec {p},X)=\min _{\vec {x} \in X}||\vec {x}-\vec {p}||. \end{aligned}$$

The closest point in *X* denoted $\vec {y}$ such that $d(\vec {p},\vec {y})=d(\vec {p},X)$. let *Y* be the resultant corresponding point set ( the set of all closest points), and let $\mathcal {C}$ be the closest point operator, then

8 $$\begin{aligned} Y=\mathcal {C}(P,X). \end{aligned}$$

The least squares registration is computed as described:

9 $$\begin{aligned} (\vec {q},d)=\mathcal {Q}(P,Y). \end{aligned}$$

The positions of the data shape point set are then updated via $P={ \vec{q} }(P)$.

**Algorithm 3.1** ICP procedure

1. Given the point set *P* with $N_p$ points ${\vec {p}}$ from the data shape and the model shape *X*.
2. The iteration is initialized by setting $P_0=P,~\vec q_0=[1,0,0,0,0,0,0]^T$ and $k=0$. The registration vectors are defined relative to the initial data set $P_0$ so that the final registration represents the complete transformation. Steps (a)–(d) in the following are applied until convergence within a tolerance $\tau$.
  - Compute the closest points: $Y_k=\mathcal {C}(P_k,X)$, where $\mathcal {C}$ denotes the closest point operator.
  - Compute the registration: $(\vec {q}_{k},d_{k})=\mathcal {Q}(P_{0},Y_{k}).$
  - Apply the registration: $P_{k+1}=\vec {q}_{k}(P_{0})$.
  - Terminate the iteration when the change in mean-square error falls below a preset positive threshold $\tau$ (i.e. $\Vert d_{k}-d_{k+1}\Vert <\tau$), which specifies the desired precision of the registration, otherwise, set k = k+1, go to step (a).

It is worth noting that in Eq. (8) $\mathcal {C}$ is not a unique map from *P* to *X*, but this does not influence the algorithm. The ICP algorithm does not require a one-to-one correspondence between *P* and *X*. It was proved in Ref. [25] that the ICP algorithm always monotonically converged to a local minimum with respect to the mean square distance objective function. Our superpositioning algorithm also works well as demonstrated in all of our numerical experiments.

### The combined procedure for pairwise and multiple structure superposition

The principal component analysis gives the principal-axes of each protein structure. The ICP algorithm is a powerful method for points registration. However, it is only converges to a local minimum value and is sensitive to the initial value. In the following, we introduce the combined procedure for the pairwise structure superposition in detail.

Data preprocessing is needed. We download proteins from the National Center for Biotechnology Information (NCBI) database or other database, and the format is **P**rotein **D**ata **B**ank (PDB). We extract 3-dimensional coordinate and put the data into txt format. The Matlab program runs on the system of windows7, with AMD Athlon(tm) P340 Dual-Core Processor.

**Algorithm 3.2** Pairwise structure superposition

1. Input the proteins structure data $P_a$, $P_b$, set initial value k = 1.
2. Employ principal component analysis to find the principal components. For each of the two proteins $P_a$ and $P_b$, the eigenvectors and eigenvalues is calculated ($u_1, u_2, u_3$ for $P_a$ and $v_1, v_2, v_3$ for $P_b$), and the geometric center is determined.
3. The protein $P_b$ is rotated. The rotating axis goes through O (0, 0, 0) (geometric center) and parallels to the vector *v* (here, *v* is $v_1$ or $v_1\underline{+}v_2$ or $v_1\underline{+}v_3$). The interval degree is set to $10^{\circ }$.
4. For each rotated position of $P_b$, the eigenvectors and eigenvalues is calculated again. The principal-axes of the new $P_b$ and $P_a$ is aligned using least square method.
5. The ICP algorithm is applied.
6. If RMSD $<c$ (e.g., $c=1.5$) or number of iterations exceeds certain times, output the cumulative rotation matrix and translation vector, break; Else, go back to 3.
7. If RMSD $>c$, (e.g., $c=1.5$) for the whole circle. Then we choose the smallest RMSD case, and output the rotation matrix and translation vector.

The multiple structures superposition algorithm is a natural extension of that for pairwise structure superposition. We first suggest to use the one with the median length of structure chains as the template protein. The key idea is applying pairwise structure superposition to calculate the superposition between the remaining proteins and the template protein. For example, there are three proteins X, Y, Z to be superimposed, and assuming protein X is the middle protein (model protein), then, Y is superimposed on structure X, Z is also superimposed on structure X.

The details of our multiple algorithm are as follows:

**Algorithm 3.3** Multiple structure superposition

1. Input the protein structures, $C={P_1, P_2,\ldots , P_n}$, $n\ge 3$.
2. Calculate the length of each protein and sort them by length.
3. Choose the middle sized protein as the template structure, denoted as $M_i$, for each protein in *C* calling the pairwise proteins superposition algorithm, output the RMSD between this protein and the template and this protein’s number, denoted as set $T_{i}$. The initial value *i* is equals 1.
4. For each protein in $T_{i}$, sort by RMSD in ascending order. If the RMSD $< c$ (e.g., $c=1.5$), we put the proteins and the corresponding RMSD in set $S_{i}$. If the RMSD $>c$, we put the proteins and the corresponding RMSD in set $T_{i+1}$.
5. Choose the largest RMSD protein in set $S_{i}$ as template $M_{i+1}$, for each protein in $T_{i+1}$ calling the pairwise proteins superposition algorithm, change RMSD in $T_{i+1}$.
6. $i \leftarrow i+1$, using step 4 and step 5, update $M_i$, $T_i$ and $S_i$.
7. If $|T_{i}|=|T_{i+1}|$ or $|T_{i}|=0$, stop.
8. Output each protein rotation matrix *R* and translation vector *T*.

### Performance metrics

There are two parameters to measure the quality of the protein structure superposition: the number of residues that are aligned in the superposition and the average pairwise root mean squared deviation (RMSD) between aligned atoms. Clearly, the goal is to minimize the RMSD while maximizing the number of residues used in the superposition. In the following sections, if we do not mention the number of points used in superposition, the number is the smaller one between a pair of proteins.

## Results

In this section, we tested our method PSSM using both simulated data and protein structures from the PDB. We compared it with several typical methods including least square (LS), $C_\alpha$-match [26], CPSARST [27], CCP4 [28], SuperPose [29] and MUSTANG [30].

### Results of the simulated data

We used the protein structure d1cih (835) as an example, and generated four rotated structures with three random rotational orthogonal matrices $r_1$, $r_2$ and $r_3$ and one specific matrix $r_4$ representing a 90-degree-rotation around z-axis.

The four rotation matrices $r_1$, $r_2$, $r_3$, $r_4$ are as follows:

$$\begin{aligned} r_1= \left[ \begin{array}{lll} -0.2579 &{} 0.8740 &{} 0.4117 \\ -0.7291 &{} 0.1035 &{} -0.6766 \\ -0.6339 &{} -0.4747 &{} 0.6106 \end{array}\right] , \end{aligned}$$

$$\begin{aligned} r_2= \left[ \begin{array}{lll} 0.1853 &{} 0.5045 &{} -0.8433 \\ 0.8945 &{} -0.4419&{} -0.0678 \\ -0.4069 &{} -0.7417 &{} -0.5332 \end{array}\right] , \end{aligned}$$

$$\begin{aligned} r_3= \left[ \begin{array}{lll} -0.6533 &{} -0.7515 &{} -0.0926 \\ 0.2860 &{} -0.3581 &{} 0.8888 \\ -0.7010 &{} 0.5541 &{} 0.4489 \end{array}\right] , \end{aligned}$$

$$\begin{aligned} r_4= \left[ \begin{array}{lll} 0 &{} -1 &{} 0 \\ 1 &{} 0 &{} 0 \\ 0 &{} 0 &{} 1 \end{array}\right] . \end{aligned}$$

We superimposed the four structures on the original one. Numerical experiments show that PSSM works well for all cases (Table 1). However, the running time is different due to the position and orientation of initial solutions to the optimal one. We can also use all possible correspondence between two structures and apply least square (LS) directly. It can also give a better superposition. The complexity of this algorithm is $O(n^2)$, where *n* is the number of sequence-aligned atoms. However, this algorithm needs sequence alignment. The complexity of our pairwise structure superposition is $O(mn)$, where *m* and *n* are the number of the C($\alpha$) atoms of the proteins.

**Table 1:** The superposition results of PSSM for two identical protein structures with one randomly generated by a rotation from another one

Structure data	Time (s)	RMSD (Å)
$v.- v.*r_1$	317.8	$4.0628*10^{-14}$
$v.- v.*r_2$	1161.4	$4.2752*10^{-14}$
$$v.- v.*r_3$$	27.3	$$ 5.0009*10^{-14}$$
$$v.- v.*r_4$$	2.3	$$ 2.0260*10^{-14}$$

Because the least square (LS) method is popular and serves as an optimality criterion for determining the best superposition, we compared our method with it. We use the $C_\alpha$ atomic coordinates of five pairwise protein structures from d1cih, d1lfma, d1m60a, d2pcbb, and d1kyow to demonstrate our method can get similar superposition accuracy with LS. We can see that our algorithm indeed get almost the same RMSD as LS (Table 2). Although PSSM may take more time, it doesn’t require the initial correspondence or sequence alignment.

**Table 2:** Comparison between PSSM and LS

Structure name	Time (s)		RMSD (Å)	
id1(size)–id2(size)	LS	PSSM	LS	LS
d1cih (108)–d1lfma (103)	0.002	0.331	0.6	0.6
d1cih (108)–d2pcbb (104)	0.007	9.418	0.7	0.7
d1cih (108)–d1m60a (104)	0.006	21.484	1.2	1.2
d2pcbb (104)–d1m60a (104)	0.007	7.394	1.3	1.3
d1cih (108)–d1kyow (108)	0.002	3.768	0.7	0.7

Numerical experiments show that our method can get comparative RMSD with larger number of aligned residues than $C_\alpha$-match and CPSARST (Table 3). This may be because our method treats the structure with missing data as a whole structure.

**Table 3:** Comparison of PSSM with $C_\alpha$-match and CPSARST

PDB/SCOP entries	PSSM		$C_\alpha$-match		CPSARST	
id1(size)–id2(size)	Aligned	RMSD (Å)	Aligned	RMSD (Å)	Aligned	RMSD (Å)
1nls (237)–2bqpA (228)	228	1.4	214	1.3	218	1.4
1glh (214)–1cpn (208)	208	0.7	206	0.5	206	0.5
1yadA (190)–2duaA (283)	190	2.6	130	1.7	151	2.4
1zbdA (177)–1pujA (261)	177	3.2	113	1.5	130	3.2
d1nkla (78)–d1qdma1 (77)	77	2.6	49	1.4	70	2.4

“Aligned” means how many residues were aligned

**Table 4:** Comparison of PSSM with CCP4 and SuperPose

PDB/SCOP entries	PSSM		CCP4		SuperPose	
id1(size)–id2(size)	Aligned	RMSD (Å)	Aligned	RMSD (Å)	Aligned	RMSD (Å)
1nls_ (237)–2bqpA (228)	228	1.4	114	1.0	205	18.1
1glh_ (214)–1cpn_ (208)	208	0.7	156	0.4	156	0.4
1yadA (190)–2duaA (283)	190	2.6	157	2.4	183	10.6
1zbdA (177)–1pujA (261)	177	3.2	97	2.0	177	20.0

“Aligned” means how many residues were aligned

We compare our PSSM method with CCP4 and SuperPose (Table 4) and find that each method has its own advantage. We adopt four pairs of proteins including 1nls and 2bqp, 1glh and 1cpn, 1yad and 2dua, 1zbd and 1puj as testing system. Take 1nls and 2bqp as an example, PSSM gets 228 aligned residues ($C_\alpha$) with RMSD of 1.4Å, CCP4 gets 114 aligned 114 residues with RMSD of 0.999Å and SuperPose gets 205 aligned residues with RMSD of 18.14Å. Compared with CCP4 and SuperPose, PSSM gets more aligned residues, and gives reasonable and competitive RMSD compared those obtained by CCP4, and demonstrates overall better results than SuperPose. A possible reason is that SuperPose uses a secondary structural alignment strategy to guide the superposition. It is proper for secondary structural alignment and good at detecting domain or hinge motions in proteins. While our method is designed for the full structure superposition (see more examples in Additional file 1: Tables S2 and S3).

We also benchmark the performance of PSSM against DALI and MATT using Fischer’s benchmark dataset (Table 5). Fischer’s dataset is a popular benchmark for testing protein structure alignment programs, and they contain 68 pairs of protein structures. In Table 5, we use the average aligned residues and the average RMSD. (The pairs alignment performance can be seen in Additional file 1: Tables S4 and S5). Table 5 shows the performance. The average RMSD (aveRMSD) of our method is greater than DALI AND MATT, but the average aligned (aveAligned) residue is longer than DALI and MATT.

**Table 5:** Performance comparison on Fischer’s dataset

Fischer’s dataset (67 of 68 pairs)	DALI	MATT	PSSM
aveAligned	155	152	186
aveRMSD (Å)	2.77	2.87	2.90

### The usability of PSSM algorithm

The following analysis show how the missing data affect the performance of PSSM. We keep one copy of a protein structure and delete some atoms from another copy of it to simulate a protein structure with missing data. Two deleting approaches are explored. The first one is deleting the atoms in order and the second one is deleting the atoms in a random way.

**Fig. 3:**
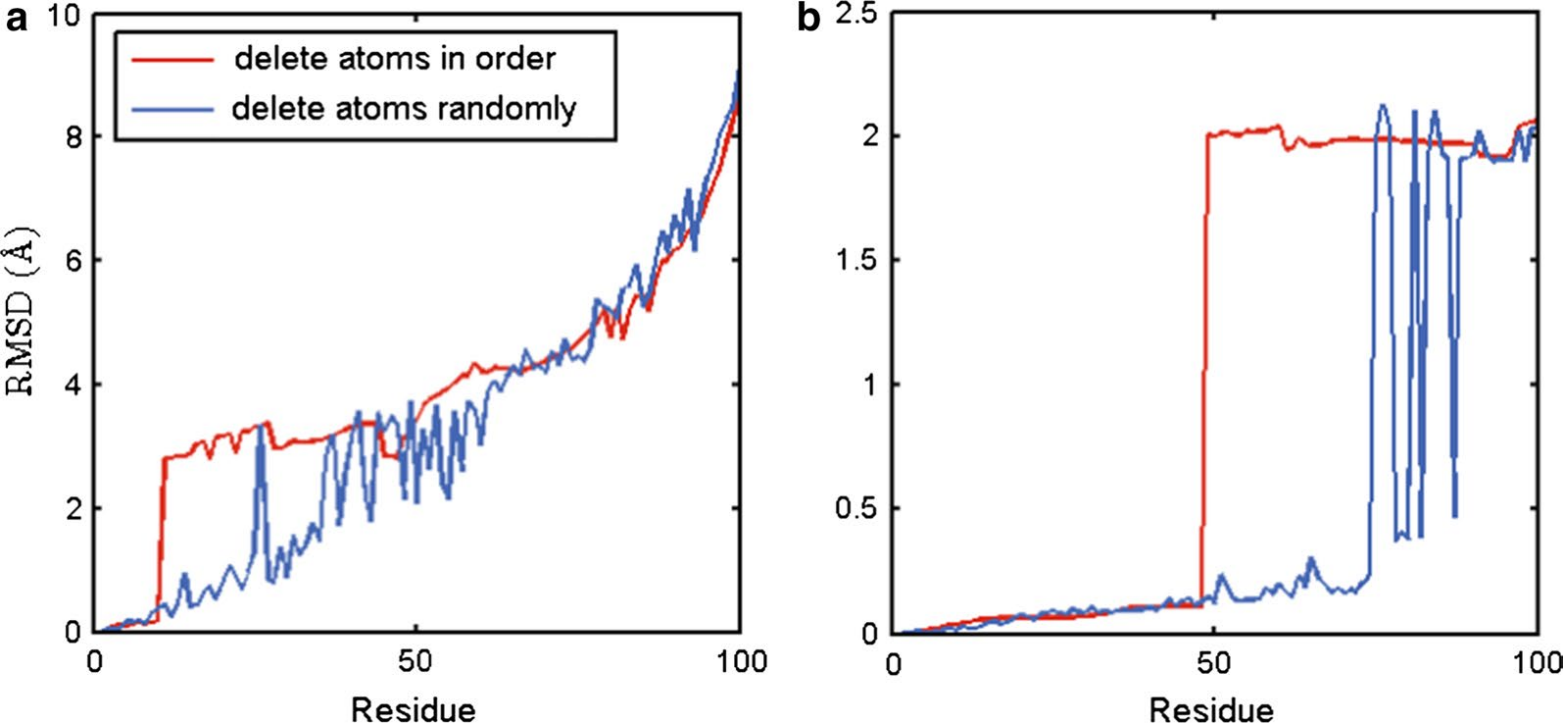
Superposition RMSD of d1cih and one of its rotated configuration with a number of deleted (**a**) $C_\alpha$ atoms or (**b**) main chain atoms, respectively. The atoms are deleted in a random (*blue color*) or an ordered manner in the first 100 atoms (*red color*), respectively

Figure 3a shows that the performance of pairwise superposition between the protein structure d1cih (with only $C_\alpha$ atoms) and a mimic structure through a rotation of d1cih. Here the rotation matrix is:

$$\begin{aligned} r= \left[ \begin{array}{lll} 0.9548 &{} 0.2182 &{} 0.2019 \\ -0.1777 &{} 0.9634 &{} -0.2007 \\ -0.2383 &{} 0.1558 &{} 0.9586 \end{array}\right] , \end{aligned}$$

d1cih has 108 $C_\alpha$). We can see that when deleting the atoms in order and the number of deleted atoms is below 10, the RMSD is very small. However, when the number is greater than 10, the RMSD is sharply increased to about $3\AA$. In this case, for random deleting, the RMSD keeps small until the deleted atoms are more than 20.

Figure 3b shows that the performance of pairwise superposition between the protein structure d1cih with all main chain atoms and a mimic structure through the rotation of d1cih. The rotation matrix *r* is:

$$\begin{aligned} r= \left[ \begin{array}{lll} 0 &{} 1 &{} 0 \\ 1 &{} 0 &{} 0 \\ 0 &{} 0 &{} 1 \end{array}\right] , \end{aligned}$$

d1cih’s main chain has 835 atoms. When deleting the atoms in order, the results show that the RMSD keeps small up to 50 atoms deleted. However, when the deleted atoms are more than 50, the RMSD is sharply increased to $2\AA$, and can keep at similar level till 70 atoms deleted.

From the above analysis, we can see that PSSM for pairwise structure superposition is relatively robust for the case with random missing data than with sequential missing data. From the two cases above and more cases we run, we find that PSSM requires the difference between the two protein lengths less than about 20 %, for structure superposition with missing data.

### Multiple protein structure superposition

We test our method using the proteins from three families. One of a ten protein structure superposition case is from the cytochrome C family which includes d1cih, d1pcbb, d1lfma, d1crj, d1csu, d1csx, d1yeb, d1kyow, d1m60a, and d1u74d. The other two families are Globins and Serine Proteinases. We choose five proteins for the Globins family and seven proteins for the Serine Proteinases. These proteins have different amino acid sequence, yet similar structures. We choose d1cih, 2dhbb and 2pka as the template protein structure which has the median length. In practice, we only need to find one template protein in the case. We show the results of our method for the pairwise superposition RMSD in these three families (Tables 5, 6, 7), respectively. We can see that our PSSM method works very well. There is only one case 2pka versus 1ppb with relatively larger RMSD than other pairs (Table 6).

**Table 6:** The RMSD of pairwise superposition between d1cih and others with PSSM for cytochrome C

PDB-id1 (size)–PDB-id2 (size)	Time (s)	RMSD (Å)
d1cih (835)–d1crj (847)	2.301	0.3829
d1cih (835)–d1csu (846)	2.685	0.3881
d1cih (835)–d1csx (846)	2.674	0.4852
d1cih (835)–d1yeb (847)	3.108	0.7979
d1cih (835)–d1kyow (850)	48.480	0.9363
d1cih (835)–d1lfma (800)	6.399	1.0420
d1cih (835)–d2pcbb (823)	424.890	1.1760
d1cih (835)–d1u74d (847)	1196.996	0.8338
d1cih (835)–d1m60a (819)	754.727	1.4786

**Table 7:** The RMSD of pairwise superposition between 2pka and others with PSSM for serine proteinases data set

PDB-id1 (size)–PDB-id2 (size)	Time (s)	RMSD (Å)
2pka (232)–3est (240)	397.0237	1.5222
2pka (232)–1ton (227)	300.4844	1.3310
2pka (232)–3rp2 (224)	460.3419	1.5825
2pka (232)–4ptp (223)	236.3811	1.1994
2pka (232)–5cha (236)	454.4466	1.7583
2pka (232)–1ppb (295)	542.5367	2.9835

**Table 8:** The RMSD of pairwise superposition between 2dhbb and others with PSSM for Globins data set

PDB-id1 (size)–PDB-id2 (size)	Time (s)	RMSD (Å)
2dhbb (146)–1hhoa (141)	26.9685	1.4944
2dhbb (146)–1hhob (146)	0.2768	1.0898
2dhbb (146)–2dhba (141)	43.1975	1.4393
2dhbb (146)–1mbd (153)	15.4869	1.4735

We also compared our PSSM with MUSTANG using five proteins in the Globins family and seven proteins in the Serine Proteinases family as testing systems (Tables 8, 9). Generally, these two methods have shown very competitive results. For the Globins family, PSSM is better than MUSTANG with two more aligned residues and even a bit smaller RMSD (1.37 versus 1.41 Å). As to the Serine Proteinases family, PSSM aligned more atoms with a slightly larger RMSD (1.72 versus 1.56 Å)

**Table 9:** Comparison of PSSM with MUSTANG using the Globins and Serine Proteinases data sets

Data sets	PDB codes	PSSM		MUSTANG	
		RMSD (Å)	Aligned	RMSD (Å)	Aligned
Globins (5)	1hhoa, 2dhba, 1hhob, 2dhbb, 1mbd	1.37	141	1.41	139
Serine Proteinases (7)	3est, 2pka, 1ton, 3rp2, 4ptp, 5cha, 1ppb	1.72	223	1.56	205

## Conclusion

We have proposed an effective method PSSM for superpositioning pairwise and multiple structures with missing data. The method does not need a sequence alignment in advance. It employs the principal component analysis to find the initial rough superposition, and then uses an iterative closest point algorithm for refining and getting accurate registration. According to what we’ve known, this is the first time to combine PCA and ICP algorithm to study the problem of non-sequential superposition. Numerical experiments demonstrate its accuracy and effectiveness. This method has the comparable accuracy as the least square method which is a classical method for protein structure superposition. However, the least square method needs the sequence alignment.

## Additional file

10.1186/s13015-016-0079-3 The superposition results of PSSM for two identical protein structures with one randomly generated by a rotation from another one. **Table S2.** The RMSD of pairwise superposition between 2pka and others with PSSM for Serine Proteinases data set|3rp2b, 1arb, 1ppb, 1sgt, 1ton, 2alp, 2sga, 2snv, 4ptp, 5chab. **Table S3.** The RMSD of pairwise superposition between 2pka and others with PSSM for Serine Proteinases data set|3rp2b, 1arb, 1ppb, 1sgt, 1ton, 2alp, 2sga, 2snv, 4ptp, 5chab. **Table S4.** The RMSD of pairwise superposition with PSSM for Fischer's dataset (67 pairs). **Table S5.** The RMSD of pairwise superposition with PSSM for Fischer's dataset (67 pairs). **Table S6.** Comparison between PCA+ICP and ICP.

## Abbreviations

3D: three-dimensional
EM: Expectation Maximization
PSSM: Protein Structure Superposition method for addressing the cases with Missing data
ICP: iterative closest point
PCA: principal component analysis
RMSD: root mean squared deviation
NCBI: National Center for Biotechnology Information
PDB: Protein Data Bank
LS: least square
CPSARST: Circular Permutation Search Aided by Ramachandran Sequential Transformation
CCP4: Collaborative Computational Project Number 4
MUSTANG: MUltiple STructural AligNment AlGorithm
CAS: Chinese Academy of Sciences
