# Supplementary material for: An effective sequence-alignment-free superpositioning of pairwise or multiple structures with missing data
Source: Algorithms Mol Biol. 2016 Jun 21;11:18. doi: 10.1186/s13015-016-0079-3 (PMC4915111; doi:10.1186/s13015-016-0079-3)
Supplement: Supplementary file 1 — 10.1186/s13015-016-0079-3 The superposition results of PSSM for two identical protein structures with one randomly generated by a rotation from another one. Table S2. The RMSD of pairwise superposition between 2pka and others with PSSM for Serine Proteinases data set|3rp2b, 1arb, 1ppb, 1sgt, 1ton, 2alp, 2sga, 2snv, 4ptp, 5chab. Table S3. The RMSD of pairwise superposition between 2pka and others with PSSM for Serine Proteinases data set|3rp2b, 1arb, 1ppb, 1sgt, 1ton, 2alp, 2sga, 2snv, 4ptp, 5chab. Table S4. The RMSD of pairwise superposition with PSSM for Fischer's dataset (67 pairs). Table S5. The RMSD of pairwise superposition with PSSM for Fischer's dataset (67 pairs). Table S6. Comparison between PCA+ICP and ICP. [file 13015_2016_79_MOESM1_ESM.pdf]

Table S1: The superposition results of PSSM for two identical protein structures with one randomly generated by a rotation from another one.

Structure data	time (s)	RMSD (Å)
$v. - v. * s_1$	29.8	$1.102 * 10^{-14}$
$v. - v. * s_2$	611.8	$1.766 * 10^{-14}$
$v. - v. * s_3$	298.4	$1.287 * 10^{-14}$
$v. - v. * s_4$	254.7	$3.032 * 10^{-14}$
$v. - v. * s_5$	294.24	$1.905 * 10^{-14}$
$v. - v. * s_6$	546.2	$2.359 * 10^{-14}$
$v. - v. * s_7$	484.1	$3.528 * 10^{-14}$
$v. - v. * s_8$	393.5	$1.131 * 10^{-14}$
$v. - v. * s_9$	342.1	$3.210 * 10^{-14}$
$v. - v. * s_{10}$	397.8	$3.093 * 10^{-14}$
$v. - v. * s_{11}$	1379.0	$2.897 * 10^{-14}$
$v. - v. * s_{12}$	440.3	$2.927 * 10^{-14}$
$v. - v. * s_{13}$	399.1	$4.269 * 10^{-14}$
$v. - v. * s_{14}$	240.1	$1.560 * 10^{-14}$
$v. - v. * s_{15}$	257.0	$1.535 * 10^{-14}$
$v. - v. * s_{16}$	2934.6	$2.083 * 10^{-14}$
$v. - v. * s_{17}$	355.0	$1.516 * 10^{-14}$
$v. - v. * s_{18}$	428.7	$2.309 * 10^{-14}$
$v. - v. * s_{19}$	554.4	$1.984 * 10^{-14}$
$v. - v. * s_{20}$	361.7	$0.959 * 10^{-14}$
$v. - v. * s_{21}$	209.4	$3.336 * 10^{-14}$
$v. - v. * s_{22}$	301.4	$1.070 * 10^{-14}$
$v. - v. * s_{23}$	531.4	$1.293 * 10^{-14}$
$v. - v. * s_{24}$	806.5	$2.064 * 10^{-14}$
$v. - v. * s_{25}$	358.2	$1.816 * 10^{-14}$
$v. - v. * s_{26}$	231.4	$1.703 * 10^{-14}$
$v. - v. * s_{27}$	327.0	$2.353 * 10^{-14}$
$v. - v. * s_{28}$	606.0	$1.102 * 10^{-14}$
$v. - v. * s_{29}$	406.5	$2.344 * 10^{-14}$
$v. - v. * s_{30}$	967.6	$1.143 * 10^{-14}$
$v. - v. * s_{31}$	28.2	$1.102 * 10^{-14}$
$v. - v. * s_{32}$	423.2	$1.324 * 10^{-14}$
$v. - v. * s_{33}$	295.8	$1.287 * 10^{-14}$

The rotation matrices are as follows:

$$s_1 = \begin{bmatrix} -0.6101 & 0.5428 & 0.5772 \\ -0.4169 & 0.3996 & -0.8164 \\ -0.6738 & -0.7387 & -0.0175 \end{bmatrix},$$

$$s_2 = \begin{bmatrix} -0.6204 & -0.2010 & -0.7581 \\ -0.5381 & -0.5941 & 0.5979 \\ -0.5706 & 0.7788 & 0.2604 \end{bmatrix},$$

$$s_3 = \begin{bmatrix} -0.4854 & 0.6825 & -0.5464 \\ -0.6778 & -0.6885 & -0.2580 \\ -0.5523 & 0.2451 & 0.7968 \end{bmatrix},$$

$$s_4 = \begin{bmatrix} 0.6735 & 0.0733 & -0.7355 \\ 0.7290 & 0.0985 & 0.6774 \\ 0.1222 & -0.9924 & 0.0129 \end{bmatrix},$$

$$s_5 = \begin{bmatrix} -0.5875 & 0.4445 & -0.6762 \\ -0.4185 & -0.8821 & -0.2163 \\ -0.6926 & 0.1559 & 0.7043 \end{bmatrix},$$

$$s_6 = \begin{bmatrix} -0.4983 & -0.8155 & -0.2944 \\ -0.1082 & -0.2784 & 0.9544 \\ -0.8602 & 0.5074 & 0.0505 \end{bmatrix},$$

$$s_7 = \begin{bmatrix} -0.5161 & 0.8095 & -0.2797 \\ -0.7387 & -0.5860 & -0.3331 \\ -0.4336 & 0.0347 & 0.9005 \end{bmatrix},$$

$$s_8 = \begin{bmatrix} -0.2253 & 0.9691 & -0.1009 \\ -0.7003 & -0.2330 & -0.6748 \\ -0.6774 & -0.0813 & 0.7311 \end{bmatrix},$$

$$s_9 = \begin{bmatrix} -0.5579 & -0.1280 & -0.8200 \\ -0.7333 & -0.3866 & 0.5593 \\ -0.3885 & 0.9133 & 0.1218 \end{bmatrix},$$

$$s_{10} = \begin{bmatrix} -0.2712 & 0.9489 & -0.1614 \\ -0.7137 & -0.3107 & -0.6278 \\ -0.6459 & -0.0551 & 0.7615 \end{bmatrix},$$

$$s_{11} = \begin{bmatrix} -0.4766 & 0.5386 & -0.6948 \\ -0.5012 & -0.8158 & -0.2886 \\ -0.7223 & 0.2106 & 0.6587 \end{bmatrix},$$

$$s_{12} = \begin{bmatrix} -0.4296 & 0.8984 & 0.0909 \\ -0.6992 & -0.2672 & -0.6631 \\ -0.5715 & -0.3484 & 0.7430 \end{bmatrix},$$

$$s_{13} = \begin{bmatrix} -0.4283 & -0.4712 & -0.7710 \\ -0.2528 & -0.7567 & 0.6029 \\ -0.8676 & 0.4531 & 0.2050 \end{bmatrix},$$

$$s_{14} = \begin{bmatrix} -0.6128 & 0.7309 & 0.3004 \\ -0.5747 & -0.1513 & -0.8043 \\ -0.5424 & -0.6655 & 0.5127 \end{bmatrix},$$

$$s_{15} = \begin{bmatrix} -0.2893 & 0.8928 & 0.3452 \\ -0.8099 & -0.0361 & -0.5855 \\ -0.5103 & -0.4489 & 0.7335 \end{bmatrix},$$

$$s_{16} = \begin{bmatrix} -0.5601 & 0.4294 & -0.7085 \\ -0.7659 & -0.5944 & 0.2453 \\ -0.3158 & 0.6800 & 0.6618 \end{bmatrix},$$

$$s_{17} = \begin{bmatrix} -0.6156 & 0.2958 & -0.7304 \\ -0.4920 & -0.8683 & 0.0629 \\ -0.6157 & 0.3981 & 0.6801 \end{bmatrix},$$

$$s_{18} = \begin{bmatrix} -0.5837 & 0.7030 & -0.4062 \\ -0.4932 & -0.7044 & -0.5104 \\ -0.6450 & -0.0976 & 0.7580 \end{bmatrix},$$

$$s_{19} = \begin{bmatrix} -0.4456 & -0.3473 & -0.8251 \\ -0.4056 & -0.7433 & 0.5320 \\ -0.7981 & 0.5717 & 0.1903 \end{bmatrix},$$

$$s_{20} = \begin{bmatrix} -0.5132 & 0.6697 & -0.5368 \\ -0.7804 & -0.6244 & -0.0329 \\ -0.3572 & 0.4020 & 0.8431 \end{bmatrix},$$

$$s_{21} = \begin{bmatrix} -0.5892 & -0.7663 & 0.2562 \\ -0.5741 & 0.6202 & 0.5345 \\ -0.5685 & 0.1678 & -0.8054 \end{bmatrix},$$

$$s_{22} = \begin{bmatrix} -0.5291 & 0.4570 & -0.7150 \\ -0.7194 & -0.6885 & 0.0923 \\ -0.4501 & 0.5632 & 0.6930 \end{bmatrix},$$

$$s_{23} = \begin{bmatrix} -0.6239 & -0.4594 & -0.6323 \\ -0.5336 & -0.3407 & 0.7741 \\ -0.5710 & 0.8203 & -0.0326 \end{bmatrix},$$

$$s_{24} = \begin{bmatrix} -0.6594 & 0.2869 & 0.6949 \\ -0.6286 & 0.2968 & -0.7189 \\ -0.4125 & -0.9108 & -0.0154 \end{bmatrix},$$

$$s_{25} = \begin{bmatrix} -0.6134 & 0.2683 & -0.7428 \\ -0.4408 & -0.8967 & 0.0401 \\ -0.6553 & 0.3520 & 0.6683 \end{bmatrix},$$

$$s_{26} = \begin{bmatrix} -0.2197 & 0.5535 & -0.8034 \\ -0.7019 & -0.6616 & -0.2638 \\ -0.6775 & 0.5060 & 0.5338 \end{bmatrix},$$

$$s_{27} = \begin{bmatrix} -0.5876 & 0.8065 & 0.0652 \\ -0.6812 & -0.4496 & -0.5777 \\ -0.4366 & -0.3839 & 0.8136 \end{bmatrix},$$

$$s_{28} = \begin{bmatrix} -0.5289 & 0.8487 & -0.0011 \\ -0.5807 & -0.3629 & -0.7288 \\ -0.6189 & -0.3848 & 0.6848 \end{bmatrix},$$

$$s_{29} = \begin{bmatrix} -0.3689 & 0.5616 & 0.7407 \\ -0.6954 & 0.3620 & -0.6208 \\ -0.6167 & -0.7440 & 0.2570 \end{bmatrix},$$

$$s_{30} = \begin{bmatrix} -0.6141 & 0.0051 & 0.7892 \\ -0.5512 & 0.7129 & -0.4335 \\ -0.5648 & -0.7013 & -0.4349 \end{bmatrix},$$

$$s_{31} = \begin{bmatrix} -0.6101 & 0.5428 & 0.5772 \\ -0.4169 & 0.3996 & -0.8164 \\ -0.6738 & -0.7387 & -0.0175 \end{bmatrix},$$

$$s_{32} = \begin{bmatrix} -0.6932 & -0.2188 & -0.6867 \\ -0.3218 & -0.7586 & 0.5666 \\ -0.6449 & 0.6137 & 0.4555 \end{bmatrix},$$

$$s_{33} = \begin{bmatrix} -0.4854 & 0.6825 & -0.5464 \\ -0.6778 & -0.6885 & -0.2580 \\ -0.5523 & 0.2451 & 0.7968 \end{bmatrix},$$

Table S2: The RMSD of pairwise superposition between 2pka and others with PSSM for Serine Proteinases data set—3rp2b, 1arb, 1ppb, 1sgt, 1ton, 2alp, 2sga, 2snv, 4ptp, 5chab.

PDB-id1 (size) - PDB-id2 (size)	time (s)	RMSD (Å)
1hbg (147) - 1dlw (116)	118.7457	2.8733
1hbg (147) - 1dly (121)	150.6759	2.8976
1hbg (147) - 1eco (136)	174.0155	2.4144
1hbg (147) - 1hhoa (141)	158.7770	1.5476
1hbg (147) - 1idra (127)	136.1807	3.0678
1hbg (147) - 1mbd (153)	173.4517	1.8758
1hbg (147) - 2dhba (141)	181.2872	1.5406
1hbg (147) - 2lh7 (153)	253.3690	1.7857
1hbg (147) - 2lhb (150)	197.8880	3.0071
2lhb (150) - 1dlw (116)	110.8473	2.8965
2lhb (150) - 1dly (121)	126.5153	2.5458
2lhb (150) - 1eco (136)	157.7282	1.9400
2lhb (150) - 1hhoa (141)	190.0480	1.6050
2lhb (150) - 1idra (127)	148.3596	3.0526
2lhb (150) - 1mbd (153)	152.4995	1.8395
2lhb (150) - 2dhba (141)	239.9263	2.2500
2lhb (150) - 2lh7 (153)	148.1381	2.6678
2lh7 (153) - 1dlw (116)	199.0244	2.5296
2lh7 (153) - 1dly (121)	191.9039	2.8006
2lh7 (153) - 1eco (136)	208.4923	1.8813
2lh7 (153)- 1hhoa (141)	184.4857	2.2181
2lh7 (153) - 1idra (127)	163.8764	2.8306
2lh7 (153) - 1mbd (153)	188.8048	2.3653
2lh7 (153) - 2dhba (141)	185.1804	2.1977
1mbd (153) - 1dlw (116)	146.2766	2.8923
1mbd (153) - 1dly (121)	177.8680	2.8537
1mbd (153) - 1eco (136)	137.9191	1.5818
1mbd (153)- 1hhoa (141)	0.6807	1.4171
1mbd (153) - 1idra (127)	160.6658	3.2068
1mbd (153) - 2dhba (141)	39.1165	1.4910
2dhba (141) - 1dlw (116)	171.5952	2.7852
2dhba (141) - 1dly (121)	218.6971	2.8004
2dhba (141) - 1eco (136)	182.2503	2.1688
2dhba (141)- 1hhoa (141)	0.3387	0.7833
2dhba (141) - 1idra (127)	147.6556	2.2387
1idra (127) - 1dlw (116)	0.5781	1.0342
1idra (127) - 1dly (121)	249.7899	2.9798
1idra (127) - 1eco (136)	165.4192	2.2767
1idra (127)- 1hhoa (141)	217.1628	2.8354
1hhoa (141) - 1dlw (116)	300.5034	2.7892
1hhoa (141) - 1dly (121)	325.0068	2.8910
1hhoa (141) - 1eco (136)	264.7433	1.8185
1eco (136) - 1dlw (116)	177.7523	2.5686
1eco (136) - 1dly (121)	275.6881	2.6954
1dly (121) - 1dlw (116)	166.5940	1.1127

Table S3: The RMSD of pairwise superposition between 2pka and others with PSSM for Serine Proteinases data set—3rp2b, 1arb, 1ppb, 1sgt, 1ton, 2alp, 2sga, 2snv, 4ptp, 5chab.

PDB-id1 (size) - PDB-id2 (size)	time (s)	RMSD (Å)
3rp2b (224) - 1arb (263)	354.0209	3.0358
3rp2b (224) - 1ppb (295)	335.5111	3.0364
3rp2b (224) - 1sgt (223)	414.6835	1.8916
3rp2b (224) - 1ton (227)	347.3929	1.5982
3rp2b (224) - 2alp (198)	277.1414	2.9432
3rp2b (224) - 2sga (181)	267.7657	2.8544
3rp2b (224) - 2snv (151)	200.6259	2.7985
3rp2b (224) - 4ptp (223)	41.6531	1.3901
3rp2b (224) - 5chab (236)	345.5080	1.8112
2alp (198) - 1arb (263)	392.7250	2.8593
2alp (198) - 1ppb (295)	506.6403	1.9858
2alp (198) - 1sgt (223)	596.2109	2.9701
2alp (198) - 1ton (227)	458.3382	2.9942
2alp (198) - 2sga (181)	0.7504	0.9375
2alp (198) - 2snv (151)	241.9369	3.1091
2alp (198) - 4ptp (223)	376.6579	2.8194
2alp (198) - 5chab (236)	288.9490	2.9463
5chab (236) - 1arb (263)	524.1428	2.8670
5chab (236) - 1ppb (295)	644.3250	2.9800
5chab (236) - 1sgt (223)	521.8615	1.6044
5chab (236) - 1ton (227)	537.3022	1.7191
5chab (236) - 2sga (181)	461.0353	2.8222
5chab (236) - 2snv (151)	442.8766	2.7086
5chab (236) - 4ptp (223)	159.7629	1.1747
2snv (151) - 1arb (263)	279.4526	2.7787
2snv (151) - 1ppb (295)	284.2037	2.6855
2snv (151) - 1sgt (223)	188.6011	1.9962
2snv (151) - 1ton (227)	330.1240	2.6562
2snv (151) - 2sga (181)	267.8850	2.3343
2snv (151) - 4ptp (223)	244.0516	2.7979
2sga (181) - 1arb (263)	216.1531	2.7375
2sga (181) - 1ppb (295)	317.6448	2.2328
2sga (181) - 1sgt (223)	230.9012	1.9027
2sga (181) - 1ton (227)	357.7779	2.6987
2sga (181) - 4ptp (223)	298.9267	2.8919
1sgt (223) - 1arb (263)	334.3098	2.6814
1sgt (223) - 1ppb (295)	405.7971	2.9891
1sgt (223) - 1ton (227)	332.8098	1.9376
1sgt (223) - 4ptp (223)	279.1388	1.6087
4ptp (223) - 1arb (263)	293.5296	2.9552
4ptp (223) - 1ppb (295)	360.4444	2.5276
4ptp (223) - 1ton (227)	62.9740	1.4281
1ton (227)- 1arb (263)	476.3270	2.8760
1ton (227) - 1ppb (295)	554.1247	2.9022
1arb (263) - 1ppb (295)	745.4384	3.0820

Table S4: The RMSD of pairwise superposition with PSSM for Fischer's dataset (67 pairs).

PDB-id1 (size) - PDB-id2 (size)	RMSD (Å)
1mdc (133) - 1ifc (131)	1.7384
1npx (447) - 3grs (461)	2.8048
1onc (103) - 7rsa (124)	1.8605
1osa (148) - 4cpv (108)	4.0698
1pfc (111) - 3hlab (99)	2.4311
2cmd (312) - 6ldh (329)	2.4377
2pna (104) - 1shaa (103)	2.7066
1bbha (262) - 2ccya (127)	3.1712
1c2ra (116) - 1ycc (108)	2.8618
1chra (370) - 2mnr (357)	1.7318
1dxtb (147) - 1hbg (147)	1.9074
2fbjl (213) - 8fabb (214)	2.3987
1gky (186) - 3adk (194)	3.6544
1hip (85) - 2hipa (71)	1.5526
2sas (185) - 2scpa (348)	3.0552
1fc1a (206) - 2fb4h (229)	3.4507
2hpda (457) - 2cpp (405)	2.7955
1aba (87) - 1ego (85)	2.7508
1eaf (243) - 4cla (213)	2.7428
2sga (181) - 5ptp (223)	2.8919
2hhma (278) - 1fbpa (316)	3.1774
1aaj (105) - 1paz (120)	2.788
5fd1 (106) - 1iqz (81)	2.7129
1isua (62) - 2hipa (71)	2.7712
1gal (581) - 3cox (500)	3.227
1caub (184) - 1caua (181)	3.5901
1hom (68) - 1lfb (77)	3.3497
1tlk (103) - 2rhe (114)	2.8876
2omf (340) - 2por (301)	3.1984
1lgaa (343) - 2cyp (293)	2.9995
4sbva (199) - 2tbva (287)	3.2834
8ilb (146) - 4fgf (124)	2.6581
1hrha (125) - 1rnh (151)	3.0844

Table S5: The RMSD of pairwise superposition with PSSM for Fischer's dataset (67 pairs).

PDB-id1 (size) - PDB-id2 (size)	RMSD (Å)
1mup (157) - 1rbp (174)	3.2823
1cpcl (172) - 1cola (197)	3.2783
2ak3a (226) - 1gky (186)	3.6623
1atna (372) - 1atr (383)	4.1188
1arb (263) - 5ptp (223)	2.9552
2pia (321) - 1fnb (296)	2.6982
3rubl (441) - 6xia (387)	4.0835
2sara (96) - 9rnt (104)	2.8944
3cd4 (178) - 2rhe (114)	2.1613
1aep (153) - 256ba (106)	2.3361
2mnr (357) - 4enl (436)	2.3234
1ltsd (103) - 2xsc (69)	2.8774
2gbp (309) - 2liv (344)	2.4376
1bbt (186) - 2plv (288)	3.9585
2mtac (147) - 1ycc (108)	2.2937
1taha (318) - 1tca (317)	3.3054
1rcb (129) - 2gmfa (121)	3.1121
1saca (204) - 2ayh (214)	3.2988
1dsba (188) - 2trxa (109)	2.2182
1stfi (98) - 1mola (94)	2.644
2afna (331) - 1aoza (552)	3.244
1fxia (96) - 1ubq (76)	2.7609
1bgeb (159) - 2gmfa (121)	2.9025
3hlab (99) - 2rhe (114)	3.1643
3chy (128) - 2fox (138)	2.9837
2azaa (129) - 1paz (120)	2.7575
1cew (108) - 1mola (94)	2.9272
1cid (177) - 2rhe (114)	3.0431
1crl (534) - 1ede (310)	3.0722
2sim (381) - 1nsba (390)	3.3931
1ten (89) - 3hhrb (195)	2.8842
1tie (166) - 4fgf (124)	2.8179
2snv (151) - 5ptp (223)	2.7979
1gp1a (432) - 2trxa (109)	2.7303

Table S6: Comparison between PCA+ICP and ICP .

	PDB-id1 - PDB-id2	time(s)	RMSD (Å)
PCA+ICP	d1cih - d1lfma (C(α))	0.200146	0.6324
ICP	d1cih - d1lfma (C(α))	0.496020	3.4152
PCA+ICP	d1cih - d1lfma	5.117784	1.0420
ICP	d1cih - d1lfma	16.636148	2.2894
PCA+ICP	d1cih - d2pcbb (C(α))	1.350884	2.8879
ICP	d1cih - d2pcbb (C(α))	0.525894	3.1722
PCA+ICP	d1cih - d2pcbb	11.628760	1.8631
ICP	d1cih - d2pcbb	15.777264	2.1102
PCA+ICP	d2pcbb - d1lfma(C(α))	0.811292	2.7852
ICP	d1cih - d2pcbb (C(α))	0.513574	3.1765
PCA+ICP	d1cih - d2pcbb	8.478054	1.8407
ICP	d1cih - d2pcbb	8.845609	1.9943
